# Radicalization Through the Lens of Situated Affectivity

**DOI:** 10.3389/fpsyg.2020.00205

**Published:** 2020-02-18

**Authors:** Hina Haq, Saad Shaheed, Achim Stephan

**Affiliations:** Institute of Cognitive Science, Osnabrück University, Osnabrück, Germany

**Keywords:** radicalization, emotions, situated affectivity, mind invasion, affective scaffolding

## Abstract

Affective bonding to radical organizations is one of the most prominent features of a recruit’s personality. To better understand how affective bonding is established during the recruitment of youth for radicalization and how it is maintained afterward, it seems promising to adopt new insights and developments from the field of situated cognition and affectivity, particularly the concepts of Affective Scaffolding, Mind Invasion, and Self-Stimulatory Loops of Affectivity (SSLA). The three notions highlight both the intended structuring of the affective bonding by the recruiting organizations and the immersive influence these settings have on the individuals. We will study the affective bonding between an individual and a radical group from two perspectives: first, from an organizational perspective, and second from a personal perspective. The first aims at understanding how extremist organizations “invade the mind” of young people, by providing carefully designed affective scaffolding: (a) during the recruitment process and (b) while being a full member of the organization. The second aims at identifying some of the affective loops which individuals who have joined the radical organization enter.

## Introduction

On April 21, 2019, people in Sri Lanka suffered from terror attacks that resulted in the death of 253 people (Safi, 2019). This incident took place after the terrorist group who call themselves the Islamic State of Iraq and Syria (ISIS) lost all its territory in Syria and Iraq, as claimed by the ground forces in March 2019 (Callimachi, 2019). However, ISIS claims responsibility for the Sri Lanka attacks with some Sri Lankan officials initially terming it ISIS’s retaliation of the terrorist attack on the Christchurch Mosques in New Zealand on March 15, 2019 (Miglani and Pal, 2019). Sri Lankan Defense Minister Ruwan Wijewardene stated that most of the terrorists involved in these attacks were well-educated people who stem from middle- or upper-middle-class families, and were financially relatively independent. This statement might speak against what many people commonly believe about suicide bombers, as it seems hard to think of a terrorist as a well-educated and financially stable person with apparently everything to live for, who is deciding to blow themselves up in a suicide attack and kill others (Bergen, 2019).

There is no simple explanation to why and how a person is radicalized to become a terrorist and suicide bomber. Radicalization is a multi-factor, multi-pathway and complex process (e.g., Taylor and Horgan, 2006; McCauley and Moskalenko, 2008; Sageman, 2008; Schmid, 2013; Hafez and Mullins, 2015). Since 2001, many models and metaphors describing the process of radicalization have been presented by various researchers in (social) psychology, mostly interpreting it as a progression over a period of time and involving different factors and dynamics in individual (lone-actor) and collective settings. Some milestones in the literature on models of radicalization come from Moghaddam (2005), Wiktorowicz (2005), and Silber and Bhatt (2007) who offer different stage models about how an individual becomes radical and eventually violent; Kruglanski (2006) highlights the role of ideology and the “Quest for Significance” (Kruglanski et al., 2009, 2014) and proposes the so-called 3N approach (need, narrative, and network) to understand what drives individuals to radicalization and violent extremism (Webber and Kruglanski, 2017). Furthermore, experts have acknowledged a variety of factors that make people vulnerable toward radicalization as individuals (micro-level), as groups (meso-level) and as societies (macro-level) (e.g., Wiktorowicz, 2005; Sageman, 2008; Kruglanski and Fishman, 2009; Schmid, 2013; Malthaner and Waldmann, 2014). Some of the factors include uncertainty in life (Hogg and Adelman, 2013; Hogg et al., 2013), collective identity problems (Moghaddam, 2012), experiencing alienation (Horgan, 2008; Wilner and Dubouloz, 2010), and grievances stemming from discrimination, stigmatization, rejection, relative deprivation, and humiliation (Moghaddam, 2005; Bindner, 2018). They are usually combined with moral outrage and feelings of revenge (e.g., McCauley and Moskalenko, 2008; Sageman, 2008; Schmid, 2013). Radical groups make use of the factors mentioned above to their advantage in order to influence their followers to build an affective bond with the group and its ideology, which results in mobilizing the recruits to act extremely and violently.

In sociology, Social Movement Theory (SMT) is an important theoretical framework which is applied to explain radicalization. SMT focuses on broader dynamics and processes of political mobilization leading to radicalization, in which individuals and groups are driven by political goals and rational agents (e.g., Della Porta, 1992; Wiktorowicz, 2005; Dalgaard-Nielsen, 2008; van Stekelenburg and Klandermans, 2010; Beck and Schoon, 2018). Social movement theory, in the context of protest events and campaigns, explain the roles of emotions, which can be generally applied to radicalization and provides an important contribution to understanding emotions in radicalization. Organizations and social movements specifically arouse and use multiple emotions in social networks that play a key role in enabling or inhibiting mobilization for their causes and also sustain a commitment to a cause (e.g., Lofland and Stark, 1965; Khawaja, 1993; Nepstad, 2004; Benski, 2010; Jasper and Owens, 2014; Van Ness and Summers-Effler, 2018).

We agree with several of the aforementioned approaches that emotions play an important role in radicalization. In particular, they are key elements in establishing a dynamic affective bonding between a radical group and its followers. It is our goal to better understand how radical groups use a variety of external tools to attract new members, retain them, and direct them to violent actions. To do so we will apply the framework of situated cognition and affectivity (see next section) which is a recent development in cognitive science. In contrast to older (internal) approaches to the human mind it explicitly comprises environmental factors as key components of (cognitive and) affective processes. When we focus on the vital role affectivity plays in the process of radicalization, we do this without downplaying other factors that drive recruitments, such as the psychological, social and political factors mentioned above. But we take it as desideratum to investigate particularly the emotional bond between radicalized persons and the groups they belong to, to investigate how such affective bonds between recruits and radical organizations develop and how this supports radicalization, which might prove to be helpful for developing counter radicalization measures.^1^ Our analysis will help to understand both how radical groups use emotions to influence people and what kind of emotional vulnerability and receptivity makes a person ready to join a radical group and stay committed to it. In that sense, the affective bonds that radical groups develop with their recruits can be accounted for from two main perspectives, that is, an organizational perspective, and a personal perspective.

From the *organizational* perspective, radical organizations structure themselves to provide affective and social platforms for young individuals to connect with the organization and its ideology. Such organizations provide their recruits with a well-developed structure and system, which facilitate them to generate positive feelings such as strength, trust, pride, and belongingness within the group, and to generate negative feelings such as hate, anger, and disgust toward an outgroup, to mention the important ones.

From the *personal* perspective, a recruit joining the organization develops an affective bonding with the organization itself. The more that the recruits are exposed to the ideology and objectives of the radical group, and the more they start relating to it, the more their emotions develop in line with the goals of the group. The same holds true for training rituals and other activities within an organization, which gives a further boost to the affective bonding. This bond keeps the recruit in a specific and long-lasting affective state, which leads them toward a deeper involvement in that organization.

In contrast to already established views that mainly address negative emotions such as humiliation, fear, hate, anger, guilt, contempt, and disgust (e.g., Lindner, 2001; Bar-Tal et al., 2007; Wright-Neville and Smith, 2009; Feddes et al., 2012; Baele et al., 2014; Matsumoto et al., 2015; Baele, 2017; van Stekelenburg, 2017), we would like to also stress the importance of positive emotions, which in combination with negative emotions play a vital role in paving the path to radicalization. A radical organization in its ideology provides its followers with various positive emotions such as hope for a better future, pride of belonging to a certain group or religion, feeling of power as being member of a strong and feared group, and love for the radical ideology and those who follow it, fostering a sense of brotherhood among the members of the group. These positive emotions serve as strong pull-factors in recruitment. The manipulation of emotions in different combinations, including both the positive and the negative, by radical groups direct the thoughts and actions of followers and recruits. Therefore, love for the radical group and hate for the outgroup goes side by side (e.g., Brewer, 1999). If we look at the case studies of radicalized individuals, positive emotions are also at work and may even occur before the rise of negative emotions which lead toward violent actions.

For example, a former fighter for ISIS named Younes (Michael) Delefortrie came back to Belgium from Syria. According to a report on a series of interviews with radicalized individuals by Speckhard et al. (2018), Delefortrie was raised by an alcoholic and violent mother. He was introduced to Islam by second-generation Moroccans settled in Belgium, having strong family ties. Delefortrie felt it appealing that the religion bans alcohol. He later joined a radical Salafi organization named “Sharia for Belgium,” where he felt encouraged to undertake complete Salafi lifestyle, including that of Jihad. Eventually he traveled to Syria in 2013 to practice Islam. However, he returned to Belgium after spending 5 weeks in Syria to escape battleground and reunite with his wife. After the return, he felt disillusioned with his troubled life in Belgium and expressed his desire to return to Syria in his interview with the authors. Delefortrie idealized the ISIS “Caliphate” and hoped for it to extend to Brussels (Yayla and Speckhard, 2016; Speckhard et al., 2018). His case illustrates how radical groups are successful in instilling hope, pride and ingroup love in their followers in the first place, and creating a strong affective bonding with their recruits by providing affective support which was not available in other societies.

Before we start analyzing the recruitment and retention strategies of radical groups, mainly the ISIS and the Taliban, and how members bond to them, we introduce the theoretical framework of situated affectivity, in particular the notions of affective scaffolding, mind invasion, and self-stimulatory loops of affectivity. These three notions help us to understand how environmental structures effect emotions in general, and, in particular, they highlight the affective settings intended by recruiting organizations, and also the immersive influence these settings have on the individuals.

## Situated Affectivity and Affective Scaffolding

In order to understand the influence of environmental structures on individuals and their reciprocal influence, philosophical accounts which are extrapolations of so-called “situated” cognition (Walter, 2014) offer a promising framework. The central thesis of situated cognition is that cognitive processing is not solely an intracranial affair, but that it can be, and often is, supported by extracranial “tools” which ease and simplify cognitive tasks. These tools can be, for example, the morphological and physiological characteristics of our body (embodied cognition), but also our embodied interactions with an appropriately structured natural, technological or social environment (embedded or extended cognition). During recent years, researchers have expanded the debate about situated cognition to the affective domain to investigate to what extent extrabodily factors contribute to our affective life—though not in the trivial sense that most of our emotions are triggered by environmental stimuli in a certain context (e.g., Griffiths and Scarantino, 2009; Colombetti, 2014; Krueger, 2014; Stephan et al., 2014; Colombetti and Krueger, 2015; Colombetti and Roberts, 2015; Wilutzky, 2015; Slaby, 2016).

The idea of environmental scaffolding,^2^ which can be traced back to the work of Lev Vygotsky, was reintroduced by Andy Clark to comprise as scaffolds all sorts of environmental structures that can be used for enabling or facilitating certain cognitive tasks (Clark, 1997). In this broad sense, it also applies to various affective processes. We will principally distinguish two different, albeit interconnected, types of affective scaffolding—ways in which our affective life is essentially a matter of more or less intimate dependencies and interdependencies between us and our natural, technological, and social environment. The first type are so-called *user-resource interactions*; they have been in the center of attention in the early contributions to situated affectivity, the second type is so-called *mind invasion* (cf. Stephan and Walter, 2020, §§ 3 and 4), which entered philosophy of emotions rather recently, whereas social psychologists studied such phenomena under different terminology before (e.g., Parkinson, 1995).

Among the *user-resource interactions*, we distinguish cases:

(a) with *unidirectional material tools for emoting*, which are used when we exploit environmental resources in order to regulate our affective life (e.g., visit places that mean something to us, choose music, furnish our apartment)—a strong and relatively permanent example would be the decision to live in a caliphate,
(b) with *strongly coupled and integrated material tools for emoting*, which are used to enact some kind of self-stimulating activity that has been set in place and maintained over time in order to move into a certain emotional process (e.g., a deeply mourning musician whose playing sets up a mutually constraining cycle of affective responding and expression)—many activities taken by members of radical groups afford such kinds of self-stimulatory affective loops (e.g., listening to nasheeds, a form of vocal music that is used to enhance hate toward an outgroup, immersing in social media supporting extreme ideology, engaging in training rituals and learning the use of weapons),
(c) with *transiently coupled social tools for emoting*, which are used when actors choose other people as “tools” for living out or regulating their emotions (e.g., aggressors who provoke others to enter into a fight, confessors who aim at relieving their sorrows through interaction with a priest)—the life of radicals offers several occasions of such a type (e.g., taking conquered women as sex slaves, giving young recruits an outlet for aggressive impulses by allowing them to punish prisoners),
(d) *affectivity instantiated by strongly coupled social systems*, when they enact certain types of emotional dynamics over and over again (e.g., old couples enacting cycles of mutual soothing and loving, or cycles of one partner sulking, the other trying to reconcile)—strong social couplings develop both internally among members of radical groups and externally via social media between members of radical groups and followers abroad,
(e) with *coupled social tools for changing the emotional mind-set*, where someone is intentionally looking for another person as a “tool” for modifying her life and mood in order to transform her affective response repertoire *in general* (e.g., seeking a psychotherapist to work with)—when, for example, someone explicitly volunteers a radical group to get orientation for changing his or her mind and life.

The various types of user-resource interactions, which in the case of radicalization support the affective bonding between a group and its members, present corresponding instantiations of the personal perspective. Often it is the group that provides a variety of possibilities for looping effects that enhance the affective bonding of members to their groups through strong social couplings. In that sense the groups perform various sorts of mind invasion.

Among the cases of *mind invasion*, we distinguish^3^ :
(f) *affective enculturation through strongly coupled social systems*, which affects all human being in early and later life spans with particular behavior styles, group norms and emotion regimes (e.g., in early childhood, peer groups, companies)—this holds true for everybody who joins a radical group, and particularly for their children,
(g) *affective transformation through social media* by providing possibilities to interact and keep in touch over long distances, but also by spreading hate speeches with all their consequences, or allowing criminal exchanges in the darknet—social media play an important role in the recruitment of new members from abroad, and in directing specific activities including the building of bombs; they also offer lone actors facilities to develop dynamic relationships with other radicals,
(h) *tools for manipulating people*, which are deliberately launched in order to diachronically modify the attitudes and the emotional set up of the members of a target group (e.g., advertising, political campaigns)—recruitment and retainment of people for extremist groups.

In particular, type (h), which corresponds to the organizational perspective, will be relevant for our analysis of the affective scaffolds provided by radical groups. Whereas some very strong instantiations of (h) might result in what is also known as brain washing, not all types of mind invasion are such cases, not even all (h)-cases. As long as the human faculty of critical thinking and distancing from certain positions is not endangered or undermined, we should not treat all kinds of mind invasion as kinds of brain washing. Even if some activities of radical groups can be treated as cases of brain washing, it does not follow that all kinds of mind invasion they provide are instantiations of brain washing; in many interactions with the group we can find volunteering aspects on the side of the members.

We now introduce the notion of mind invasion in more detail by first following Slaby, who coined the term, and then use this perspective to present what extremist groups provide to invade the mind of recruits and members.^4^ Hereafter, we offer several tools that are used as affective scaffolds by the extremist groups to successfully invade the mind of their targets.

## Mind Invasion in Radicalization

Slaby (2016) illustrates the concept of “Mind Invasion” with the example of an intern at her new workplace. She has to learn a lot about the activities in her new work environment, how the colleagues interact on different work-related hierarchies, and how they address each other. She learns how showing certain characteristics in the workplace benefits her, and how exhibiting other characteristics does not. She eventually habituates herself to become a professional in her field and progresses to become a full-time employee where she adheres to the workplace culture in a more natural manner. Something which might have felt alien when she first joined the organization now feels natural to her: for example, the long hours at work, continually staying online, answering e-mails even after office hours, feeling anxiety of falling behind on work-related communications even outside the office hours, the ways in which one is expected to show enthusiasm and eagerness for the work, etc. The organization, as Slaby terms it, “hacks the mind” of the new employee, to the extent that she will come to feel as a part of the organization. Her habits and ambitions merge with, and change, according to the organization’s objectives^5^.

According to Slaby (2016), organizations structure themselves in a way that influences the affective setup of the individuals working within it; some organizations do this intentionally, while others create this affective environment accidentally. Humans who are inclined to think that they are in pretty good control of their affective life can, therefore, easily underestimate the power of the environment on their emotional repertoire. As it becomes evident from the examples Slaby presents, individuals working within an organizational setup can have their subjectivity hacked through the invasion of their minds by the structuring of the organization.

For the context of radicalization, it is important to note that environmental structures can be shaped by radical organizations (as tools for mind invasion), to gradually transform the attitudes and the emotional responses of a target group. Accordingly, via social media radical groups attract followers and influence the mindset of individuals [by (g)], to not only join the group but also to be drawn to their more extreme and radical ideas. In that sense, mind invasion facilitates what is described as Identity Fusion (Swann et al., 2009; Paredes et al., 2018), the phenomenon of an individual feeling “fused” or “one” with a group, and it plays a major role in the structuring of recruitment and retention strategies of radical groups. One of such recruitment strategies is called *the funnel* (Gerwehr and Daly, 2006). As the term indicates, potential recruits are funneled through a carefully structured transformative process (see (e) and (h) above). They emerge from the process as dedicated members of the radical group. This carefully structured and intended process consists of fusing the identity (Swann et al., 2009) of the recruit with the radical group, hazing rituals, commitment to the radical ideology, and support for achieving the desired goals through violence. These processes result in transforming attitudes and radical polarization in those who successfully follow the whole process along the lines as defined by the radical groups. Not all potential recruits follow the whole process. Even if they drop out before they complete the entire process, the minds of the drop-out recruits may still have been invaded to the extent that these recruits will provide benefits to the radical group through, for example, endorsing a positive image of the group among other potential recruits and even navigate others to enter the process of radicalization [according to (f)].

Mind invasion by radical groups is not limited to attract and retain new members. Radical groups spread their ideology and shape vulnerable individuals to adopt the extremist world-view in many ways. For example, in some areas of Pakistan and Afghanistan, the radical group who calls itself Taliban has access to influence people directly via giving religious sermons or running the religious educational institutes, more commonly called Madrassah (pl. Madaris). This access gives recruiters a platform to directly invade minds during early phases of enculturation (see f) with the extreme ideology and narrative which is based on hatred and anger toward the outgroup. One instance of spreading violence-based ideology was the use of an elementary Urdu language book, which was covertly being used as a part of the syllabus in some local (and low-income) schools in Pakistan. These books were also meant to be sent to Madaris and schools in the north-western region of Pakistan which had some radical ideological presence. The book was published between 2005 and 2010. Unlike the English alphabet books where the letter A is represented by an apple and B by a ball, the content of the elementary Urdu alphabets book (being taught to very young children), however, are all about implanting radical jihadi imagery in the young minds. For example, the word and image used to explain the Urdu alphabet 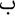 “*bey*,“consists of an illustration of a Kalashnikov and the word “*bandooq*” (gun). For the letter 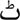 “*tay*,” the word used is “*takrao*” (impact) and an illustration of a plane hitting the Twin Towers in New York is shown. For the letter 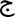 “*jeem*,” an image of a white jihadi flag and the word “jihad” is used. For the letter 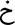 “*khay*,” an image of a hunting knife (with blood dripping from it) and the word “*khanjar*” (knife) is used. For the letter 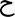 “*hey*,” an image of a woman fully covered in black cloth and the word “*hijab*” is used. For the letter 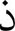 “*zey*,” the word used is “*zunoob*” (sin) and the illustration is that of a bonfire made from a pile containing a TV set, a satellite dish, a board game, and a guitar (Paracha, 2011).

Education and curricula based on extreme ideology aim to expose and normalize children to categorical thinking and violence, and results in an us-versus-them mindset. Through repeated lessons based on the extreme syllabus taught in some of the Madaris, the Taliban intentionally immerse children into an environment that teaches them hatred, anger, and disgust toward the outgroup (those who do not follow their interpretation of religion, and are perceived as threat to the sanctity of the religion). In these environments, a child is introduced to the alphabet letter 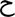 “*hey*,” an image of a woman fully covered in black cloth and the word “*hijab*” along with an illustration of a woman covered completely in black cloths. For the child, the only acceptable way for a woman is when she dressed in a hijab, and any other form of dressing is not acceptable. Along with this kind of radical educational material, the child is also provided with the explanation of how it is sinful for a woman to not properly cover herself, and that she deserves to be punished if she refuses to wear a hijab. Through the Taliban’s interpretation of Islamic law, a woman showing her face to a man who is not related to her is the source of corruption (Gohari, 2000). In a similar manner, the burning image of a guitar in fire is introduced to the child with the word “*zunoob*” (sin). The child is taught that it is not only bad to listen to or play guitar (or any other kind of music), but rather that the music is a forbidden and punishable *sin*. This kind of early-age introduction to the radical concepts create a different kind of reality within the minds of children undergoing education in radical organizations. It implies that forcing someone to wear hijab or punishing someone for listening to or playing music is the only acceptable way to live a life. This early enculturation changes the way a child looks at the world.

The same holds for the concept of Jihad. The *radical milieu* (Malthaner and Waldmann, 2014) makes it natural for the child to adopt extremist views. When the child grows up, he or she might not join a radical group, but the radical group has already invaded the child’s mind to the extent that he or she accepts and/or supports the extremist ideology. It can also be seen in the children getting education in ISIS territories (e.g., Olidort, 2016). Children who are exposed to this kind of education might not yet be recruits of the radical group, but their lifestyles and ambitions are already developing in alignment with the parameters of the radical group’s ideology (e.g., Al Bayan Center, 2016; Anderson, 2016; NCTV and AIVD, 2017), which raises additional problems for the countries their parents come from, if they return with their mothers or families and join schools in Western countries.

Different radical groups use different strategies to shape vulnerable individuals, depending on their organizational structure and aims. Where the Taliban mainly use their easy access to the religious schools and mosques as a tool of mind invasion, ISIS uses more diverse environmental tools to invade the minds of its followers. One such strategy of ISIS is using different social platforms to create an environment which helps them to spread their ideology [see (g) and (h)]. They *invade the minds* of young people by showing them a glorified and adventurous face of ISIS and by repeatedly providing its followers glimpses of its ideology through various means, including its mass media propaganda (e.g., Winter, 2015b). The use of social media, nasheeds, and video games, for example, as tools of mind invasion, over time facilitates the transfer of followers from a virtual caliphate to an active recruit either as fighter in ISIS claimed territories or as “lone actor” fighters in other countries. For example, one Birmingham-born ISIS member compared his so-called Jihad in Syria with a famous first-person shooter video game, “Call of Duty”by tweeting; “*you can sit at home and play call of duty or you can come here and respond to the real call of duty. The choice is yours*” (Kang, 2014). A British foreign fighter of ISIS tweeted that “*It’s actually quite fun. It’s really really fun. It’s better than that game Call of Duty. It’s like that but it’s in 3D where everything is happening in front of you*” (Klausen, 2015).

These tweets with the references of famous video games are reinforced by statements from people who have experienced life under ISIS and describe how adventurous their life seems to be. ISIS uses video games as an *external tool* to brand itself as being a fun and adventurous organization to join. Through social media postings like these, ISIS members portray the image of the radical group as a powerful and “*cool*” organization (Vale, 2018). To a young individual looking for orientation, this might look like living the life of a hero participating in a real-life adventure and saving the oppressed from infidels. This also provides a chance to follow an ideology which can transform their prosaic life into a cosmic struggle between good and evil (Kang, 2014). Mind invasion works because it distracts with one thing (for instance, as being a *hero*) while accomplishing another (that is guiding toward violent radicalization). In addition, ISIS strategically approached young people by providing them a platform to redress the grievances they hold against an outgroup, as well as play their part in building an Islamic community. It provided an opportunity to become a hero and a chance to achieve glory while fighting in a real-life *game*. With *mind-invading* strategies like these (e.g., Lakomy, 2019), potential recruits willingly go through the change because of the excitement to be actively part of the action and play the real-time “Call of Duty.” At some point, radicalizable individuals are attracted to radical groups because of the promise of adventure (e.g., Speckhard and Ahkmedova, 2006; Vale, 2018), but the structure of the organization aligns the recruit’s ambitions with the group’s ideology later in the radicalization process.

While training, new recruits are exposed to literature and videos that paint a vivid picture of Muslim suffering and humiliation by the hands of infidels [see (f)]. The perceived narrative of humiliation by the outgroup (infidels) aggregates the emotions of anger and contempt in recruits, and shames them into taking revenge for their Muslim brothers (Hafez and Mullins, 2015).

Another strategy which ISIS uses to shape the minds of its current and potential recruits is by evoking the sense of competition (Vale, 2018). For instance, ISIS regularly publishes a segment under the title of “Hasaad ul Ajnaad (Harvest of the Soldiers)” in its magazines and videos (e.g., Bilger, 2014; Cafarella et al., 2019). Harvest of the Soldiers informs the ISIS followers of the number of successful attacks and operations conducted. These details show the damages and losses they have caused to the outgroup. On the one hand, this propaganda is intended to keep the morale of its followers high by showing how powerful ISIS is. On the other hand, it increases the sense of competition among the recruits in different areas which were controlled by ISIS. By simply publishing the numbers of attacks, ISIS triggers different emotions, like power and pride, which aim at motivating more violent attacks to achieve higher ranks in the within group competition.

Another example of reaching out to followers [by (g)] is when ISIS captured a Jordanian pilot on December 24, 2014, and ISIS started a campaign on Twitter to decide how to punish this pilot. The pilot was burned alive and run over by a bulldozer in response to an online competition which allowed members, associates, and even passive followers of ISIS to suggest different ways in which the pilot could be executed. Thousands of people used the Arabic Twitter hashtags to suggest that Moaz (the pilot) be cut into pieces by a chainsaw, fed to crocodiles, impaled, set on fire, and even being mutilated using acupuncture needles dipped in acid before being beheaded and having the head sent back to Jordan. A second hashtag carried even more ruthless execution ideas. It was re-tweeted more than 11,000 times (Richards, 2015; The Carter Center, 2015).

This latter example shows us how ISIS strategically used social media to attract thousands of minds from around the world to participate in a ruthless crime (e.g., Blaker, 2015; Alava et al., 2017). Not all the people who participated in it were radicalized or members of ISIS. However, ISIS appealed to the minds of its followers and recruits by turning a brutal act into an online competition and people participated in dehumanizing another human being without even completely considering what they were doing. The mind invasion performed by the radical group was not limited to their active recruits but also aimed at their sympathizers.

## Specific Tools Provided for Mind Invasion

As indicated in the preceding section, radical organizations use multiple recruitment mechanisms: potential members are sometimes radicalized individually, and sometimes in groups. In this section, we highlight three important ways in which ISIS uses ideological appeals, social appeals, and material appeals as affective scaffolds to invade and radicalize vulnerable minds.

On an *ideological* level, radical organizations provide their followers with a strong narrative appealing to the vulnerabilities of the potential recruits (Macnair, 2018; Macnair and Frank, 2018). The radical organization almost always use narratives as affective scaffolds to attract its followers. In places where there is a sense of deprivation, radical organizations almost always present and propagate the narrative of religion in their lands and among themselves, mixing it with the political narrative of their own. The narrative which radical organizations present is one that is familiar and extremely trusted among the followers because of its sacred value derived from ancient religious roots (Atran and Axelrod, 2008; Ginges et al., 2009). Therefore, many are receptive to this narrative and are also easily manipulated by it (Boutz et al., 2019).

In the case of ISIS, once it declared itself a “caliphate” in June 2014, it attracted many followers, not only from Iraq and Syria, but also from abroad (e.g., Soufan Group, 2015). ISIS focused on increasing its influence by gaining territory and by calling on Muslims to perform “Hijrah” (migration) to join their newly established caliphate, where sharia law would be practiced (Schmid, 2015). The concept of “Hijrah” is sacred for many Muslims because of its religious significance and history. Therefore, the invitation of calling the Muslims to perform Hijrah held considerable appeal and had aroused the spiritual feelings in not only the followers of ISIS but also those who wished to live under the Islamic law and saw Hijrah to ISIS “caliphate” as a religious obligation (Bin Sudiman, 2017). According to Hogg and Adelman (2013), radical groups provide the vulnerable and potential recruits with a particular identity and give the recruits meaning to their lives. Radical groups lessen the uncertainty of potential followers by telling them how they would feel and behave within the group, and toward the outgroup. The group also instructs on what they should think and how they should retaliate if the ideology becomes threatened (Hogg and Adelman, 2013; Ugarriza and Craig, 2013).

Bloom and Horgan (2019) illustrate instances in which terrorist organizations, specifically those following *jihadi* narratives, structure themselves to support the culture of martyrdom. Martyrdom is a revered concept among many Muslims in general because it implies the sacrifice of human life for the sake of protection of the religion as a sacred duty and offers honor, opportunities to regain significance, fame and respect (e.g., Bloom, 2005; Güss et al., 2007; Kruglanski et al., 2009; Bélanger et al., 2014; Speckhard and Shajkovci, 2019). In the case of violent jihadi radicals, it explains the motivation of becoming a fighter or volunteering for suicide bombings. The sanctity of martyrdom shows that material incentives do not necessarily matter for all its recruits. For some, the narrative of the highest rank in heaven could be the only spiritual goal. As Bloom and Horgan (2019) report, for younger male suicide bombers, the incentive is of virgins in heaven, along with respect in this world. For younger girls, the incentives based on the jihadi narrative is that they will receive more beauty and will have seventy of their closest relatives join them in heaven, thus allowing the feeling of helping their loved ones and family in the afterlife.

In the communities where there is chaos because of war or violence, radical groups can provide potential recruits with order and identity. Taking control of the caliphate by ISIS provides its potential recruits with an environment (territory) which facilitates and nurtures the recruits’ religious and spiritual feelings. The new caliphate claim provides its followers with the hope of getting their existential needs answered (e.g., Cottee and Hayward, 2011; McBride, 2011). The caliphate allows a unity of Muslims under the sharia law and the protection of Islam against the threat of the outgroup. It should be noted that the calls to join ISIS through its propaganda were not *only* the narrative of hate and fight against the outgroup, but also the promise of living in a land that will be based on (their interpretation of) the Islamic principles. The organization provides its followers with a trusted and familiar picture of the law set up in their “claimed” lands. The narrative also paints the picture that during the rule of ISIS, individuals would be able to design their Islamic lifestyle, not only on the individual level but also on a collective level (Winter, 2015a).

We now move on to explain how affective scaffolding is established and used to invade minds on the *social* level. Radicalization requires a feeling of familiarity, trust, and commitment between the radical organization and its recruits, as it supports clear communication and emotional shaping. The most secure source for recruitment of radical members is from their own families, friends, and like-minded activists within already existing setups (Hafez, 2016), also termed as “Kinship Recruitment.” According to Hafez, kinship recruitment links individuals who share similar beliefs, which helps in creating an immediate collective identity. Kinship recruitment provides the familiar and trusted atmosphere for both the recruiter and the potential-recruit, in which both can attain their desired affective states [see (b) and (d)]. Kinship recruitment also leads to the formation of a bloc within a recruited group, known as bloc recruitment, which refers to the recruitment of multiple individuals from the same family or group. The bloc serves another advantage to the radical organization because it makes it easier for individuals within the bloc to accept radical ideas. We stress again that the affectivity in radicalization is not necessarily based *only* on violent emotions. The affective scaffolds blocs and groups provide for individuals support the feelings of religious pietism, brotherhood, and belongingness. Radical groups such as ISIS and the Taliban, infuse their group’s ideologies with their interpretation of religion (e.g., Al-Ansari and Hasan, 2018). Such ideologies are easily accepted by their followers and recruits. Any threat toward the radical group is then also considered a threat toward the sanctity of the religion. Therefore, protection of the collective identity against the threat of an outgroup becomes a salient feature of the group and is justified (e.g., Paredes et al., 2018).

On the *material* level, the recruits are provided with incentives for playing their part for the organization. After the “caliphate” claim, the ISIS leader in June 2014, called out to Muslim professionals such as doctors, engineers, and people with military and administrative expertise to join ISIS (Site Intelligence Group, 2014). Some joined ISIS in response to this call, with the intention of helping Muslims in need there. Others did not join because of the ideology, but rather because of job opportunities (Harissi, 2015). According to Anderson (2016), ISIS provides its fighters with salaries and other incentives, such as supplies, gasoline, and women to marry, which helps the organization to retain its recruits and boost their morale. Children are offered gifts and toys as compensation for their loyalty to the group. There were even reports by locals of earning a salary as high as $200 per month for child recruits, which offers the family the opportunity at a better quality of life in the war and violence-torn country. These monetary incentives were usually distributed during events where there were booths displaying and distributing ISIS propaganda (Anderson, 2016).

Bloom and Horgan (2019) illustrate other strategies that utilize incentives to radicalize and recruit. One strategy used by ISIS is to make membership for children look desirable and build an atmosphere of competition for member status. Based on the competition, the children were presented with new uniforms and their pictures taken for the propaganda. For adult recruits in ISIS, women were used as a commodity. Women who pledge their allegiance to ISIS are used as potential wives for local and foreign fighters, the purpose of which was to recruit, reward and retain the fighters. Women who were captured and did not pledge their allegiance to ISIS are used as sex slaves, and are considered to be “spoils of war.” These incentives serve as scaffolds for the recruits to voluntarily join the radical ideology.

The above examples illustrate how affective bonds are deliberately created by the organization, and what particular scaffolds they use for being successful. Ideological, psychological, and material incentives and motivations created by ISIS, in our view, are directly aimed at building trust and familiarity among its followers, and at the same time providing a sense that the individuals are rewarded by the organization, thus forging a bond with its recruits and followers. In the next section, we analyze how the members of ISIS form and maintain loops of affectivity that support to keep themselves in their desired affective states within the organization, thereby focusing more on the personal perspective.

## Self-Stimulatory Loops of Affectivity in Radicalization

Self-stimulatory loops of affectivity (SSLA) refer to strong interactions between individuals and their resource [see (b) and (d)], which, when maintained over some time and place, evolve into a form of self-stimulatory activity. The idea of the self-stimulatory loop was coined by Clark (2008) with reference to cognitive tasks. Stronger types of affective individual-resource interactions have been discussed by Colombetti and Roberts (2015). Most examples discussed thus far in the literature relate, however, a user with some technical device as, for example, a musical instrument. It is important to see that strong couplings between users and resources do not only exist toward technical devices, but can also be established with other human beings, or—in the case of mind invasion—with devices, practices, etc., provided by a mind-invading group.

To see how self-stimulatory affective loops also contribute to the recruitment of new members to radical organizations, let us look at bloc recruitment. If some individuals of a particular family make a commitment to an ideology, it facilitates the commitment of other members of the same family to follow. The elements that are self-stimulating in the bloc are peer pressure within the bloc, group-thinking, the desire to maintain relationships, and feelings of guilt for staying behind (Hafez and Mullins, 2015).

When recruits join ISIS, they enter an atmosphere of specific emotions such of love and hope within the group; and hatred, anger, contempt, and disgust toward the outgroup. Within ISIS environment, the recruits (including women and children) are assigned specific roles (e.g., De Leede et al., 2017; Almohammad, 2018; Darden, 2019). The more effectively they perform their duties (e.g., executing prisoners), the more authority and power they can get, and the more empowered they feel. This SSLA pattern becomes reinforced and this loop of power results in deeper involvement of members within the organization.

Radical organizations also provide the platform which helps to keep the concerned emotions of its recruits within the SSLA. Habitual development of rituals, routines, and training also result in specific affective states for the recruits. According to the propaganda materials by ISIS, the routine of a fighter includes dedicating time to prayers, physical fitness, and activity, learning from religious texts, as well as learning combat and warfare techniques. This routine and training cycle keep the emotions of recruited fighters elevated throughout their attachment with the ISIS.

The “bridge-burning” practice, such as recruits burning their government-issued passports to signify loyalty and allegiance to ISIS, is also an instance of SSLA. This diminishes the chance of deserting the group (Hafez and Mullins, 2015). Another instance of bridge-burning, as reported by Hafez and Mullins (2015), is the camera-recorded declaration of the intention to launch an attack. By declaring allegiance to a group and intention of carrying out an act of violence, the recruits bind themselves to such an act and feelings associated with it. If a recruit backs off, he is considered a traitor and has to face humiliation among his comrades. Bridge-burning practices, therefore, keep the recruits in a specific SSLA and prevent recruits from entertaining any thoughts of leaving ISIS. Such practices and rituals also support the emotions that the recruits are all-in and prepared to do anything for the protection of ISIS.

ISIS and its leaders encourage isolation of its members from those who oppose the views of the organization and make an effort to deepening the commitment of their members to the organization. The leaders discourage their recruits to access the mass media of the “outgroup” as well, which, for its members, is the propaganda. The members have access to the materials provided solely by ISIS itself, which keeps certain affective states in a loop, such as anger, hate, and disgust [see (h)]. Web technologies provide a perfect platform for the members of ISIS to design their newsfeeds, not only as affective scaffolds but also as SSLA. The news shared by ISIS and their followers show the suffering of Muslims all over the world, creating further hate, anger, contempt, and disgust toward the outgroup. The more they follow such news, the more negative emotions they feel. ISIS uses their elevated negative emotions to help its recruits to plan more violent attacks against the outgroup. This material also motivates the recruits to become heroes of the religion (Hafez and Mullins, 2015).

Another resource provided by ISIS is the propaganda material published and aired from time to time, including its newsletters, videos, and audio recordings in the form of speeches of leaders and nasheeds (Islamic hymns). These evoke the hope of living in a caliphate and a desire to help those who are already living there. The propaganda glorifies martyrdom and is also focused on the dehumanization of the outgroup. An interesting aspect in this propaganda is the use of numbers in the newsletters and videos, specifically the “Hasaad ul Ajnaad (Harvest of the Soldiers)” reports which indicate the attack metrics of ISIS, e.g., they tell which subgroup killed how many infidels (Bilger, 2014; Cafarella et al., 2019). This report not only serves as mind invasion but also as SSLA of competition, designed to motivate its followers, inspire them and keep the fear elevated among the outgroup.

For lone actor terrorists, SSLAs can also develop as they intentionally subscribe to virtual environments projecting hate, anger, and disgust toward an outgroup. Virtual environments, facilitated by virtual networking with other extremists in chat rooms, such as that of ISIS’s “virtual caliphate” specifically in the Telegram social network website, can ultimately lead to mobilization of members toward radicalization (Bloom et al., 2019). We find similar patterns in right wing terrorism, where affectively structured virtual environments lead vulnerable minds toward developing loops of negative emotions, which eventually lead them toward violent actions, such as Tarrant’s attack in New Zealand on March 15, 2019.

An example of Bangladeshi immigrant Roshonara Choudhry, from the report of Speckhard and Shajkovci (2018), shows how SSLA works in so called lone actors. She was a student at Kings College London. She came across the sermon of Anwar al Awlaki (Yemeni-American preacher and recruiter for Al-Qaeda) on internet [see (g)] and she started relating to Awlaki’s radical ideas of individual Jihad and his anger against United States coalition’s invasion of Iraq. She actively searched for his videos and started withdrawing from the “unbelievers” around her [see (e)]. She developed her own affective loop of hate against infidels and how western countries are discriminating Muslims. She even quit university on the claim that one of her school departments is wrongly supporting Israeli brutality against Palestine. After conducting research on which local parliamentarians voted for the Iraq war, she performed her “jihad” by stabbing Labor MP Stephen Timms in his office, twice. This example shows how a person developed her own SSLA. She related to the sermons of Awlaki urging revenge, hate and anger against so-called enemies. She on purpose started avoiding people around her to feed her loops of anger and hate in isolation to the extent that it led her to attempt murder. In this particular case, none of the radical organizations was involved, nor was she contacted by any terrorist organization or their members. In many other lone actor cases, however, radical groups are in contact with the actors, and use media as an environmental tool to spread their messages and manipulate vulnerable minds to act according to the group’s ideology. They invade the minds to the extent that the person develops the desired affective loops own their own.

The careful manipulation of environmental tools by radical groups to generating customized SSLAs explain how recruits are retained in the group or are inclined toward the group’s ideology, while maintaining the desired emotional state which helps them to form a strong affective bond with the group and its ideology.

## Conclusion

Radicalization is a multi-factor process, and one of the most important of these factors is the affective aspect of radicalization. To understand radicalization, it is important to understand how radical groups or radical organizations manipulate affective aspects of human nature and convince their recruits to willingly commit brutal and violent acts of terrorism. This manipulation of human emotions is not restricted to negative emotions such as anger, disgust, but also includes positive emotions, including power, love, feelings of belongingness. During the process of radicalization, different emotions are addressed in a recruit at the same time; for instance, hate toward an outgroup and love for the ingroup fostering the feelings of belongingness and power within the radical group. Such emotions evolve with time and create a very complex emotional web. In this paper, we have tried to provide the first glimpse of how the framework of situated affectivity (especially the notions of affective scaffolding, mind invasion, and self-stimulatory loops of affectivity) can help us to understand the affective structuring of radicalization. The framework of situated affectivity highlights how radical groups provide affective scaffolding, work on the minds of their recruits, and help recruits to develop certain emotions, which deeply entrench individuals into the ideology of a radical group. The framework of situated affectivity provides a broader range of view on the affective aspects involved in radicalization. It also helps us to understand how radical groups create an environment around their potential recruits that determines who and what these potential recruits will be when they go through continuous, reciprocal, dynamical interactions with the radical groups and its affective environment. Detailing the affective processes give us an opportunity to target those environmental factors and tools which radical organizations are using to lure vulnerable minds toward the violent extremism, and helps in developing counter radicalization measures.

## Ethics Statement

Written informed consent was not obtained from the individual(s) for the publication of any potentially identifiable images or data included in this article.

## Author Contributions

All authors contributed to outline this contribution and were in charge of subsequent revisions. HH and SS wrote the manuscript, except the section “Situated affectivity and Affective Scaffolding,” which was written by AS.

## Conflict of Interest

The authors declare that the research was conducted in the absence of any commercial or financial relationships that could be construed as a potential conflict of interest.

## Footnotes

1 This move finds backing also by research in industrial-organizational psychology, where researchers have found that affective commitment, which is the strongest type of bonding, supports an enduring attachment to a company (Allen and Meyer, 1990; Mercurio, 2015).

2 For those already familiar with the framework of situated cognition and affectivity, it might suffice to say that we use the term affective scaffolding instead of “embedded” and “extended affectivity,” the distinction of which we no longer take to be promising in empirical contexts (cf. Stephan, 2018; Stephan and Walter, 2020). Our move is also supported by Kim Sterelny’s claim that the most plausible cases for the extended mind hypothesis are merely “limiting special cases of scaffolded minds,” thus depriving the extended mind paradigm of its heuristic potential, since it “obscures rather than highlights both the continuities and the differences amongst external resources and their contributions to cognitive competence” (Sterelny, 2010, p. 473).

3 Feature (g) *affective transformation through social media* is not independent from features (f) and (h); it can be part of *affective enculturation* and it can be used for *manipulating people*; we introduce it here as a separate feature for analytical reasons.

4 For those coming from situated affectivity the concept of mind invasion is useful, because it shifts the attention away from the user/resource model and it focuses instead on how the user can be “used” (or “invaded”) by the resource (or scaffold). On the other hand, scholars coming from sociology or psychology (who therefore are familiar with concepts such as enculturation) benefit from mind invasion, because this concept correctly frames the environment as something made of scaffolds and niches.

5 Although what Slaby introduces here as mind invasion, is often referred to as “company socialization” in industrial-organizational psychology (Cooper-Thomas and Anderson, 2006), the term “company socialization” could not easily be extrapolated to cover cases of recruitment and retainment as organized by radical groups.
